# RETRACTED: Wentz et al. Is There an Overlap Between Eating Disorders and Neurodevelopmental Disorders in Children with Obesity? *Nutrients* 2019, *11*, 2496

**DOI:** 10.3390/nu17101623

**Published:** 2025-05-09

**Authors:** Elisabet Wentz, Anna Björk, Jovanna Dahlgren

**Affiliations:** 1 Department of Psychiatry and Neurochemistry, Institute of Neuroscience and Physiology, University of Gothenburg, 40530 Gothenburg, Sweden; 2 Department of Pediatrics, Institute of Clinical Sciences, University of Gothenburg, 40530 Gothenburg, Sweden; anna.bjork.2@gu.se (A.B.); jovanna.dahlgren@gu.se (J.D.)

The *Nutrients* Editorial Office retracts the article, “Is There an Overlap Between Eating Disorders and Neurodevelopmental Disorders in Children with Obesity?” [1], cited above.

Following publication, the authors contacted the Editorial Office regarding the incorrect presentation of the EDE-Q results. Due to an error, the adolescent version of the EDE-Q had been administered, but the results were interpreted as if the participants had completed the EDE-Q. Therefore, the results regarding the EDE-Q instrument (including the assessment of Loss of Control Eating) are incorrect.

Given the scale and impact that this error has on the validity of the overall findings, the Editorial Board has decided that a correction would not appropriately address the scope of this issue, nor address the impact on the overall findings. Consequently, the Editorial Board has decided to retract this article [1] as per MDPI’s retraction policy (https://www.mdpi.com/ethics#_bookmark30, accessed on 11 December 2024).

This retraction was approved by the Editors-in-Chief of *Nutrients*.

The authors have agreed to this retraction. 
